# LncRNA landscape and associated ceRNA network in placental villus of unexplained recurrent spontaneous abortion

**DOI:** 10.1186/s12958-023-01107-4

**Published:** 2023-06-20

**Authors:** Minyue Tang, Qingfang Li, Shan Wan, Qingqing Chen, Shujun Feng, Jiali You, Wei Wang, Yimin Zhu

**Affiliations:** 1 Department of Reproductive Endocrinology, Women’s Hospital, School of Medicine, Zhejiang University, Hangzhou, China; 2 Key Laboratory of Women’s Reproductive Health of Zhejiang Province, School of Medicine, Women’s Hospital, Zhejiang University, Hangzhou, China

**Keywords:** LncRNA, Recurrent spontaneous abortion, Trophoblast, CeRNA, Proliferation, Apoptosis

## Abstract

**Background:**

Unexplained recurrent spontaneous abortion (URSA) is one of the most challenging conditions frustrates women of childbearing age profoundly. The gene expression patterns and biological characteristics of placental villus in patients with URSA remain largely unknown. The aim of our study was to identify potential lncRNAs as well as their action mechanisms in URSA.

**Method:**

The ceRNA microarray was used to identify the mRNA and lncRNA expression profiles of URSA patients and normal pregnancy. Functional enrichment analyses for differentially expressed mRNAs in URSA were performed. Protein-protein interaction analysis of differentially expressed mRNAs was performed to identify hub genes and key modules. Subsequently, the co-dysregulated ceRNA network of URSA was established, and the enrichment analyses for the mRNAs in the ceRNA network was implemented. qRT-PCR was performed to validated the expression of key ENST00000429019 and mRNAs in URSA.

**Results:**

We found that URSA placental villus have distinct mRNA and lncRNA expression profiles through ceRNA microarray, with a total of 347 mRNAs and 361 lncRNAs differentially expressed compared with controls. The functional enrichment analysis revealed that ncRNA processing, DNA replication, cell cycle, apoptosis, cytokine-mediated signaling pathway, ECM-receptor interaction were the potentially disrupted pathways in URSA patients. Then we constructed a co-dysregulated ceRNA network and found differentially expressed mRNAs were regulated by a small fraction of hub lncRNAs. Finally, we found a key network of ENST00000429019 and three cell proliferation or apoptosis related key mRNAs (CDCA3, KIFC1, NCAPH), and validated their expression and regulation in tissue and cellular levels.

**Conclusions:**

This study identified a key ceRNA network, which might take part in URSA and correlate with cell proliferation and apoptosis. Optimistically, this study may deepen our apprehensions about the underlying molecular and biological causes of URSA and provide an important theoretical basis for future therapeutic strategies for patients with URSA.

**Supplementary Information:**

The online version contains supplementary material available at 10.1186/s12958-023-01107-4.

## Introduction

Recurrent spontaneous abortion (RSA) is a pregnancy complication that occurs in approximately 5% of couples that refers to two or more failed clinical pregnancies [1, 2]. Multiple etiologies have been proposed for RSA including chromosomal abnormalities, infectious disease, thrombosis, endocrine dysfunction, environmental factors, and immunological factors [3]. However, the pathogenesis of RSA in 30–40% of RSA cases is not fully understood and lacks effective treatment methods, which are termed unexplained RSA (URSA) [3]. In normal pregnancy, trophoblast cells positively contribute to implantation and placentation, which is essential for the crosstalk at the fetal-maternal interface in a healthy pregnancy. Various studies have highlighted dysfunction of trophoblast as a leading contributor to this condition [4]. The inappropriate invasion or apoptosis of trophoblast cells can increase the risk of miscarriage, pre-eclampsia and fetal growth restriction [5, 6]. Moreover, some literatures also have supported the idea that trophoblast gene expression profiles of patients with URSA may be altered [4]. However, the exact mechanisms that lead to URSA are not yet fully apprehended and need further elucidation. Given the importance of trophoblast cells in pregnancy process, a deeper understanding of its molecular biology and signaling pathways changes in trophoblast cells may shed light on the etiology of URSA.

Long non-coding RNA (lncRNA) is a class of non-coding RNA with RNA transcripts of more than 200 nucleotides in length that lack protein-coding functions [7]. Many researches have indicated that lncRNAs exert their biological functions of physiological and pathological cellular conditions in a variety of human diseases [8, 9]. Competitive endogenous RNA (ceRNA) was first proposed by Salmena et al. and was defined as a class of noncoding RNAs that bind to miRNA [10]. The perturbation of ceRNA crosstalk will disrupt the balance of cellular processes and functions, leading to development of diseases [11, 12].

Recently, increasing studies suggested that multiple lncRNAs are dysregulated in reproductive diseases. For instance, lncRNA EPB41L4A-AS1 contributes to metabolic reprogramming in trophoblast cells of miscarriage [13]. Li, T et al. revealed distinct mRNA and lncRNA expression profiles of decidual natural killer cells in early missed abortion [14]. Fan et al. reported that LncRNA NEAT1 regulates apoptosis of human placental trophoblasts via miR-18a-5p axis [15]. However, there is little evidence regarding the global expression landscape of lncRNAs and mRNA in placental villus of URSA. Here, we assumed that the establishment of the lncRNA-associated network could help us to boost our apprehension about the involved processes in the URSA.

**Fig. 1:**
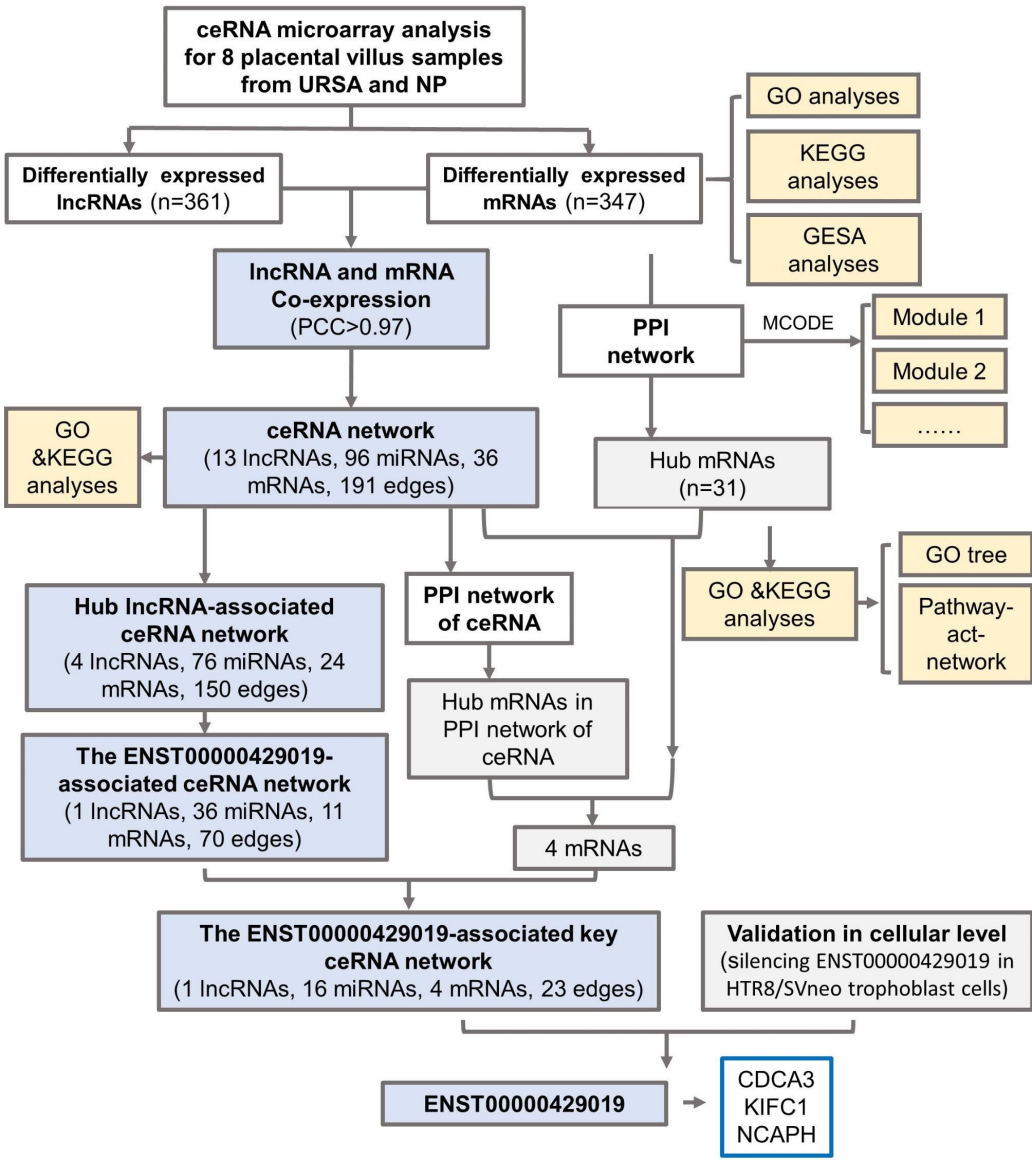
The workflow

To explore the pathological mechanism of URSA, and dig diagnostic and therapeutic value of lncRNAs in trophoblast cells, we tried to investigate the role of lncRNAs and their potential mechanisms in URSA following this workflow (Fig. 1). We gathered the expression profiles of mRNAs and lncRNA in URSA patients by ceRNA microarray. Bioinformatics analyses, including protein-protein interactions (PPI), Gene Ontology (GO) and Kyoto Encyclopedia of Genes and Genomes (KEGG) analyses were performed to identify the potential roles of the differentially expressed mRNAs (dif-mRNAs) and lncRNAs (dif-lncRNAs). Besides, co-dysregulated lncRNA-miRNA-mRNA network analysis were established and key ceRNA subnetworks may play a role in the pathogenesis of URSA were identified. In addition, qRT-PCR were used to validate the expression of hub mRNAs and lncRNA. The present work could provide novel insights into the underlying molecular processes of URSA, providing novel diagnostic biomarkers and therapeutic targets for further studies.

## Method

### Patients and tissue collection

The placenta villus tissue samples were obtained from normal pregnant (NP) women undergoing elective abortions and patients with URSA in the first trimester in the Women’s Hospital, Zhejiang University, School of medicine. Detailed patient information is summarized in the Table S1. URSA was diagnosed as two or more losses of a clinically established intrauterine pregnancy. Patients with risk factors for abortions, such as chromosomal abnormalities, abnormal maternal reproductive tract anatomy, endocrine or metabolic diseases, autoimmune disease, and so forth were excluded. All tissues were placed immediately in liquid nitrogen after removal from patients and stored at -80℃ until use. Samples from four normal pregnancy and four patients with early URSA were used for ceRNA array. The remaining samples were used for real-time PCR analysis to validate. This study was approved by the Ethical Review Committee of Women’s Hospital, School of Medicine, Zhejiang University, and all patients provided informed consent in accordance with the ethics guidelines.

### RNA isolation and microarray analysis

Placenta villus tissue samples from four URSA and four NP patients were analyzed using the human ceRNA microarray. Total RNA was isolated using the TRIzol reagent (Invitrogen, Grand Island, NY, USA). By using the Agilent Bioanalyzer 2100 (Agilent technologies, Santa Clara, CA, US), the total RNA was checked to confirm the integrity of the RNA. The microarray hybridization and collection of data were performed following a protocol from Agilent Technologies Inc., Shanghai Biotechnology Corporation.

### Functional annotation & enrichment analysis

Gene Ontology (GO) annotation and Kyoto Encyclopedia of Genes and Genomes (KEGG) pathway enrichment analysis were conducted to investigate the roles of all differentially expressed mRNAs (dif-mRNAs). GO enrichment analysis and KEGG enrichment analysis were carried out using clusterProfiler. GO-Tree was constructed to summarize the affected functions. The mutual regulation relationships between enriched KEGG pathways were illustrated by pathway-act networks. Gene Set Enrichment Analysis (GSEA) was performed using GSEAplot to evaluate the mRNAs at the level of gene sets from the Molecular Signatures Database (MSigDB).

### The establishment of the PPI network and hub genes identification

To further investigate the function of dif-mRNAs at the protein level, we constructed a PPI network by STRING (https://string-db.org/) and visualized by Cytoscape 3.8.0. Interactions with a combine score ≥ 0.7 were considered statistically significant. Then to recognize highly interacted hub mRNA clustering, we established “Molecular Complex Detection” (MCODE), a clustering algorithm identifying locally densely connected regions in a large PPI network based on node-weighting arithmetic with degree cutoff score = 2, k-core = 2 and max depth = 100. The proteins with the highest degree of connection were in the central site, and they might be most closely associated with pathogenesis. The cytoHubba app in Cytoscape was used to disclose the hub genes in the PPI network. In the whole PPI network, the top 31 hub genes ranked by degree of connectivity (degree > 20) were obtained. In the PPI network of dif-mRNAs in ceRNA work, the dif-mRNAs with degree ≥ 4 were considered to be hub mRNA.

### Construction the co-expression of lncRNA and mRNA

To explore the interactions between dif-lncRNAs and dif-mRNAs, co-expression networks were constructed in the URSA and NP samples (fold changes ≥ 2.0 or ≤ 0.5 and p-values < 0.05). We constructed the lncRNAs- mRNAs co-expression network based on the Pearson correlation coefficient (PCC) between the expression levels of mRNA and lncRNA. The value of parameter PCC > 0.97 and p-value < 0.01 was applied as a criterion to confirm the co-expression relationships [16].

### Construction of the LncRNA–miRNA–mRNA network and key subnetworks

The predicted lncRNA-miRNA pairs and miRNA-mRNA pairs were collected from databases of miRanda and TargetScan 7.1. The mRNA targets in the lncRNA- miRNA-mRNA interactions were merged with co-expression lncRNA-mRNA pairs to obtain the co-dysregulated lncRNA- miRNA-mRNA networks and were visualized by Cytoscape. After that, Gene functional enrichment and PPI analyses were performed for mRNAs in ceRNA networks.

The lncRNAs with high node degree (> 20) were chosen as hub lncRNAs, which were used with their related miRNAs and mRNAs in the regulation network to construct the subnetworks using the Cytoscape software. Then, the top-ranked lncRNAs in degree were chosen and its miRNA-mRNA pairs were extracted from the global triple network to construct core ceRNA network. The mRNAs involved in this core ceRNA network were merged with hub genes in ceRNA-PPI and whole PPI. Finally, four mRNAs were obtained as core mRNAs.

### Real-time quantitative PCR analysis

Total RNA derived from trophoblast cells or placental villus was reverse transcribed into cDNA using the PrimeScript RT Reagent Kit with gDNA Eraser (Takara Bio Inc, Shiga, Japan). The differential expression of key dif-mRNAs and one key lncRNA was validated by real-time PCR using SYBR Premix Ex Taq II (Takara Bio Inc, Shiga, Japan). The expression levels were normalized to GAPDH and analyzed using the 2-ΔΔCt method. Sequences of the specific real-time PCR primers for mRNAs and lncRNA were listed in Table S2.

### Cell culture and transfection

The human trophoblast cell line HTR8/SVneo was cultured in DMEM (Life Technologies) with 10% FBS (Gibco), 100 U/mL penicillin, and 100 mg/mL streptomycin at 37℃ and 5% CO_2_in a humidified atmosphere. We designed two siRNA for ENST00000429019 and chose the more optimal one for the following experiments (GenePharama, Shanghai, China). Transfection of small interfering RNA (siRNA) was conducted using the Lipofectamine™ RNAiMAX (ThermoFisher, Shanghai, China) according to the manufacturer’s protocol. The siRNA sequences for ENST00000429019 were showed in supplementary Table 3.

### Statistical analysis

All the experiments were repeated three times. Data analysis was performed by GraphPad Prism version 6 (GraphPad Software Inc, San Diego, CA, USA). All data were appeared as mean ± standard error of the mean (SEM). The Mann-Whitney *U* test was performed to compare mRNA and lncRNA expression between groups. Statistical significance was defined as *P*-value < 0.05.

## Results

### Identification of differentially expressed lncRNAs and mRNAs in URSA

**Fig. 2:**
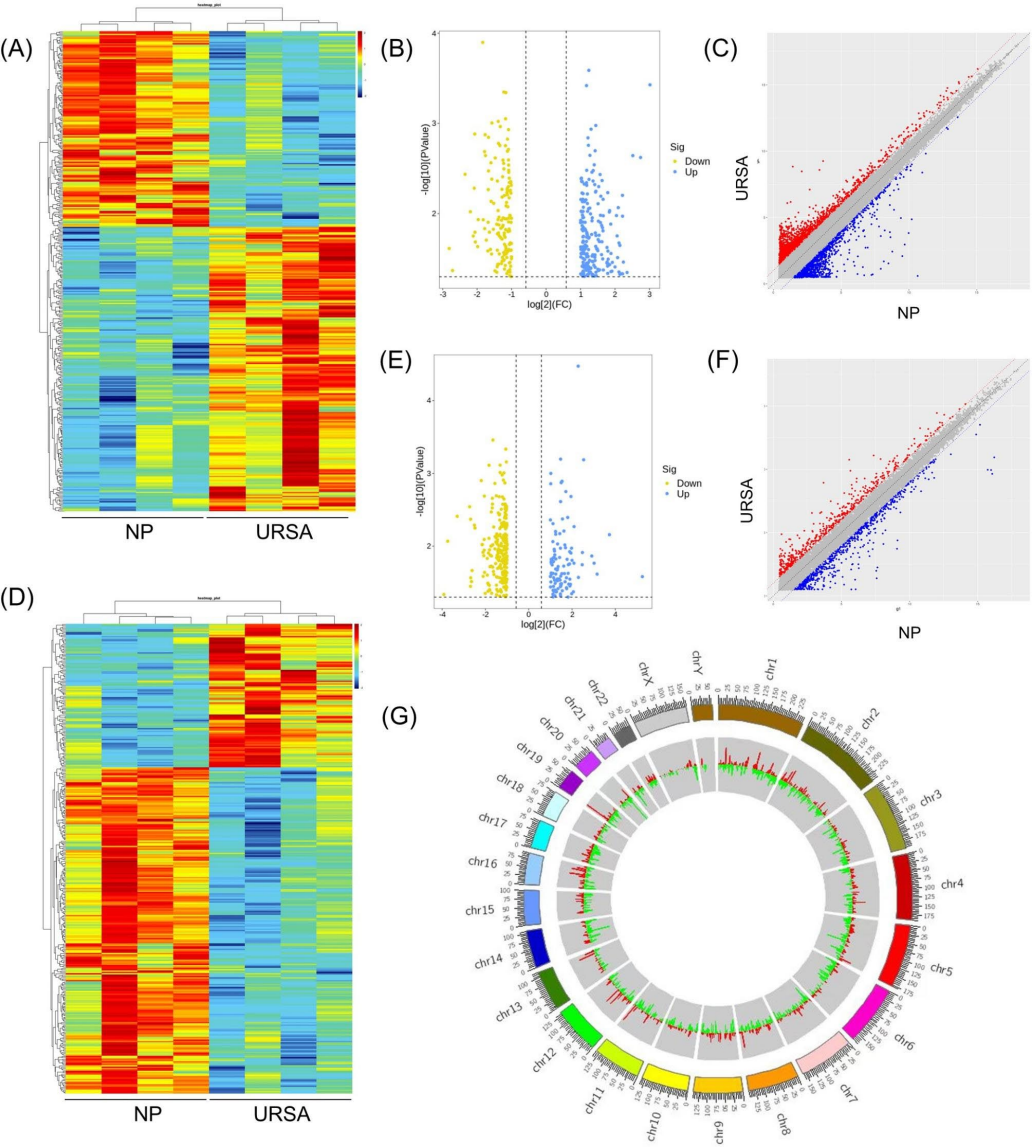
The altered lncRNA and mRNA expression profiles of placental villus inpatients with URSA. **(A)** The cluster analysis (heatmaps) of differentially expressed lncRNAs (dif-lncRNAs). **(B)** The volcano plots of dif-lncRNAs. **(C)** The scatter plots of dif-lncRNAs. **(D)** The cluster analysis (heatmaps) of differentially expressed mRNAs (dif-mRNAs). Red and blue indicate up- and downregulation, respectively. **(E)** The volcano plots of dif-mRNAs. **(F)** The scatter plots of dif-mRNAs. Red and blue indicate up- and downregulation, respectively. **(G)** The circos describing the landscape of significantly altered DELs on human chromosomes

To gain insights into the lncRNA and mRNA expression pattern and function, we performed the study as the workflow showed in Fig. 1. With the cut-off criteria of fold change ≥ 2.0 and p value *<* 0.05, a total of 361 lncRNAs (214 upregulated and 147 downregulated), and 347 mRNAs (106 upregulated and 241 downregulated) were detected as differentially expressed in villus of patients with URSA compared with control. The expression of these dif-lncRNAs and dif-mRNAs are visualized in a heatmap, scatter plot, volcano plots (Fig. 2A-F). We found that the URSA samples can be significantly separated from the control samples, indicating that the results of the differential expression analysis were reliable. The significantly altered lncRNAs were widely distributed in most chromosomes, as shown in Fig. 2G.

### Functional enrichment analysis of differentially expressed mRNAs

**Fig. 3:**
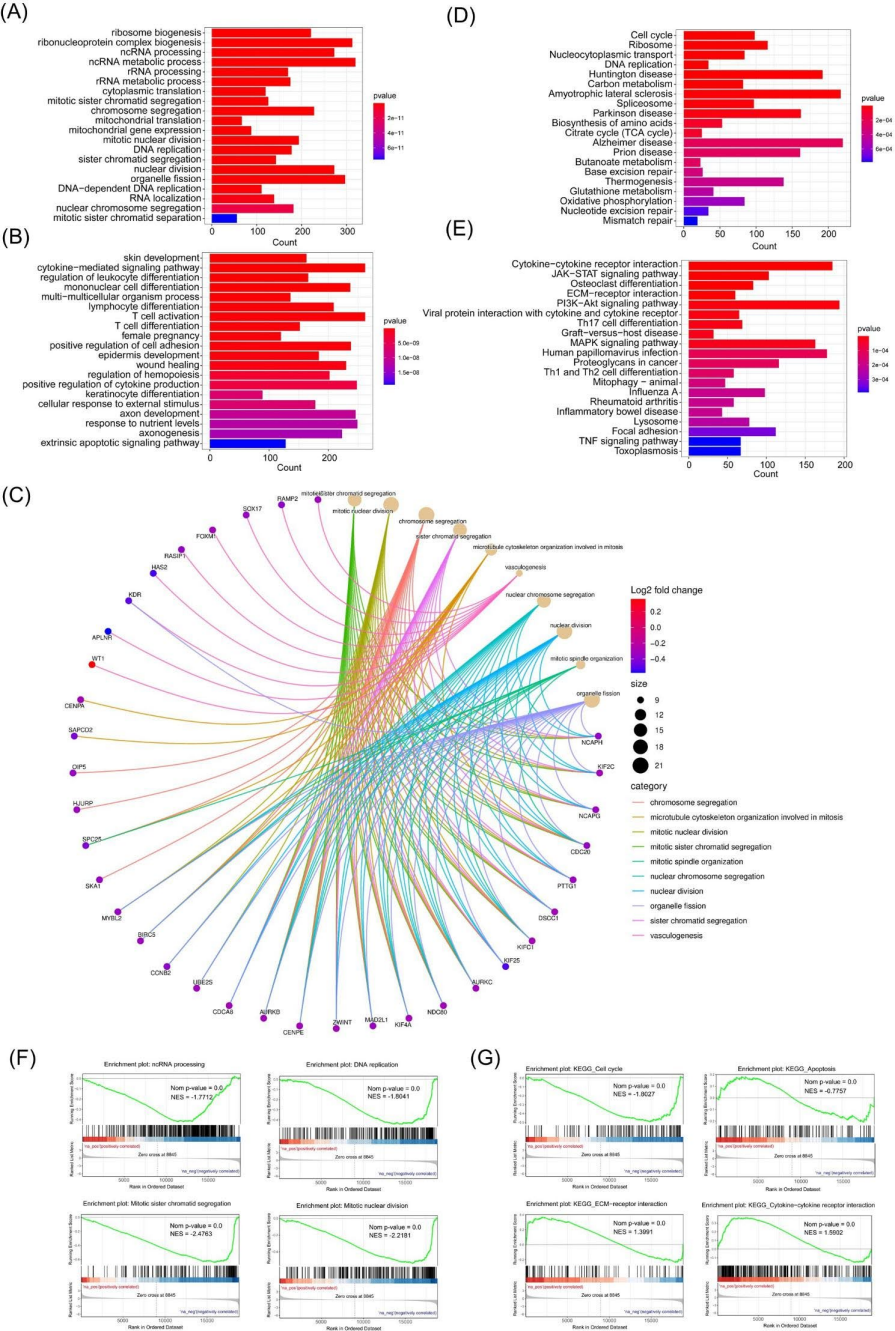
Functional enrichment analysis of differentially expressed mRNAs. **(A)** The top 20 enriched GO terms in biological processes (BP) of downregulated dif-mRNAs. **(B)** The top 20 enriched GO terms in BP of upregulated dif-mRNAs. **(C)** The top 20 enriched KEGG pathway analyses process of downregulated dif-mRNAs. **(D)** The top 20 enriched KEGG pathway analyses process of upregulated dif-mRNAs. **(E)** The top 10 enriched GO terms of all dif-mRNAs in BP and associated dif-mRNAs were showed in chord diagram. **(F)** Dif-mRNAs typically involved in the GO terms of ncRNA process, DNA replication, mitotic sister chromatid segregation, and mitotic DNA replication. **(G)** GSEA showed dif-mRNAs in KEGG annotations for cell cycle, apoptosis, ECM-receptor interaction and cytokine-cytokine receptor interaction

GO enrichment analysis and KEGG pathway analysis were performed for dif-mRNAs between URSA and NP. GO analysis results showed that changes in biological processes (BP) of downregulated dif-mRNAs were significantly enriched in ncRNA processing, ncRNA metabolic process, and DNA replication (Fig. 3A). GO analysis results showed that changes in BPs of dif-mRNAs were significantly enriched in cytokine-mediated signaling pathway, female pregnancy and extrinsic apoptotic signaling pathway (Fig. 3B). The chord diagram showed the dif-mRNAs involved in the top 10 enriched GO terms in BP, such as BIRC5, KIFC1, NCAPH, CCNB2, CDCA8 (Fig. 3C). GO-Tree analysis of BP GO terms was also performed to show their interaction relationship (Figure S1A). GO analysis results showed that changes in cell component (CC) and molecular function (MF) were enriched in condensed chromosome, centromeric region, and microtubule binding (Figure S2).

KEGG pathway analysis revealed that the downregulated dif-mRNAs were mainly enriched in cell cycle, DNA replication, nucleocytoplasmic transport ribosome (Fig. 3D), while the upregulated dif-mRNAs were mainly enriched in cytokine-cytokine receptor interaction, ECM-receptor interaction, JAK-STAT signaling pathway (Fig. 3E).

The results of GO and KEGG analysis were further confirmed and supplemented by GSEA. Dif-mRNAs typically involved in the GO terms of ncRNA process, DNA replication, mitotic sister chromatid segregation, and mitotic DNA replication (Fig. 3F). GSEA using KEGG annotations indicated that dif-mRNAs were enriched in cell cycle, apoptosis, ECM-receptor interaction and cytokine-cytokine receptor interaction (Fig. 3G). Our results suggest that trophoblasts from different groups play critical and unique roles in the pathogenesis of URSA.

### Construction of PPI networks and identification of hub genes

**Fig. 4:**
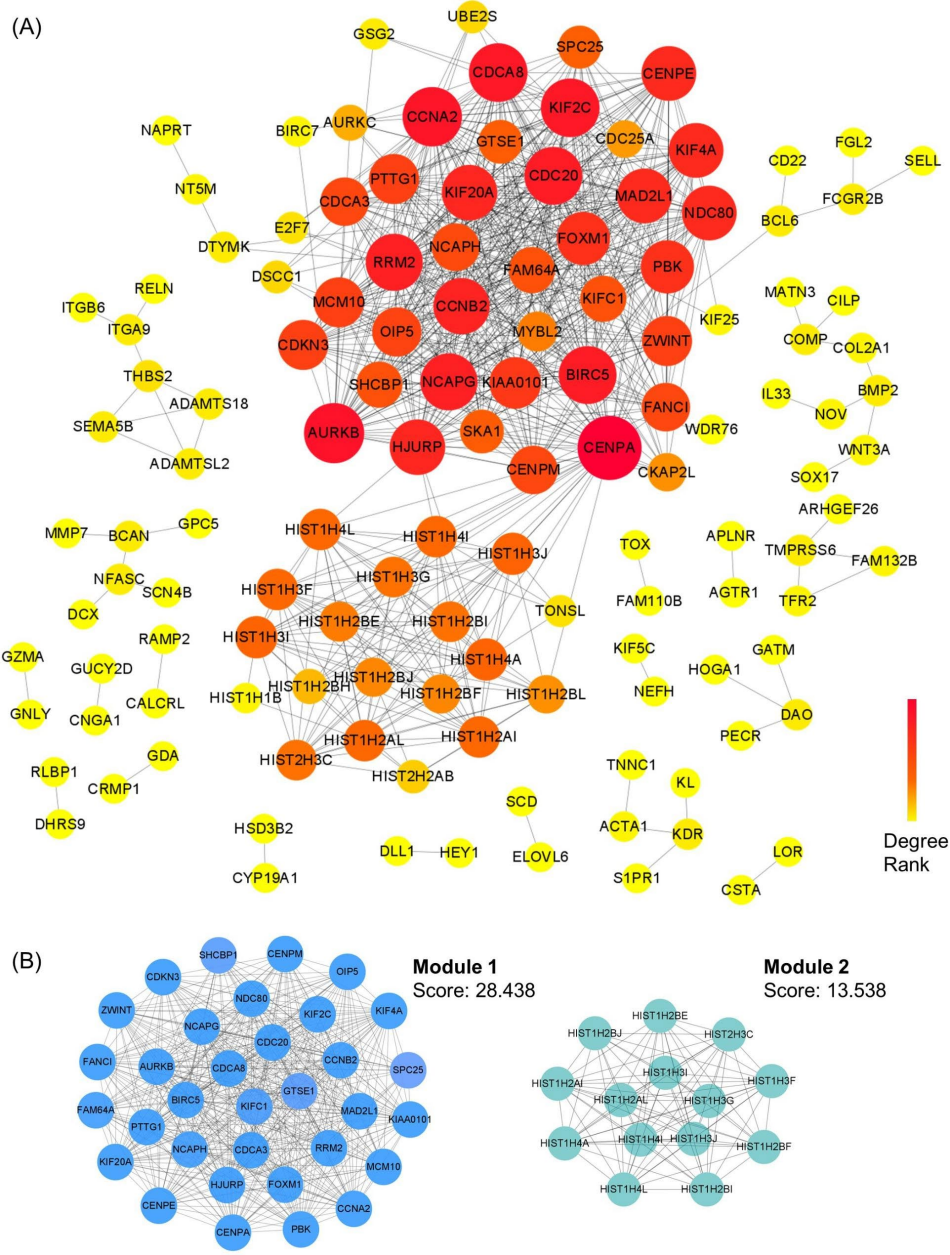
PPI network analysis of differentially expressed mRNAs and identification of hub mRNAs. **(A)** The PPI interaction network of dif-mRNAs that differed in URSA. Each edge links two interacting genes. The size and the color depth of each node correlates positively with its degree of connectivity. **(B)** The top 2 significant modules were obtained from the PPI network

To further investigate the function of dif-mRNAs at the protein level and explore the core dif-mRNAs involved in the cellular process of URSA, we constructed a PPI network with interaction score > 0.7 by STRING (Fig. 4A). The node size and color depth were positively correlated with its connectivity degree. Further, we used Cytoscape plug-in MCODE to discover densely connected regions in the PPI network. The top 2 sub-PPI network modules were shown in Fig. 4B. Module 1 consisted of 33 genes, while module 2 consisted of 14 genes from histone gene family. The functional enrichment analysis of 33 genes in module 1 were performed (Figure S3).

**Fig. 5:**
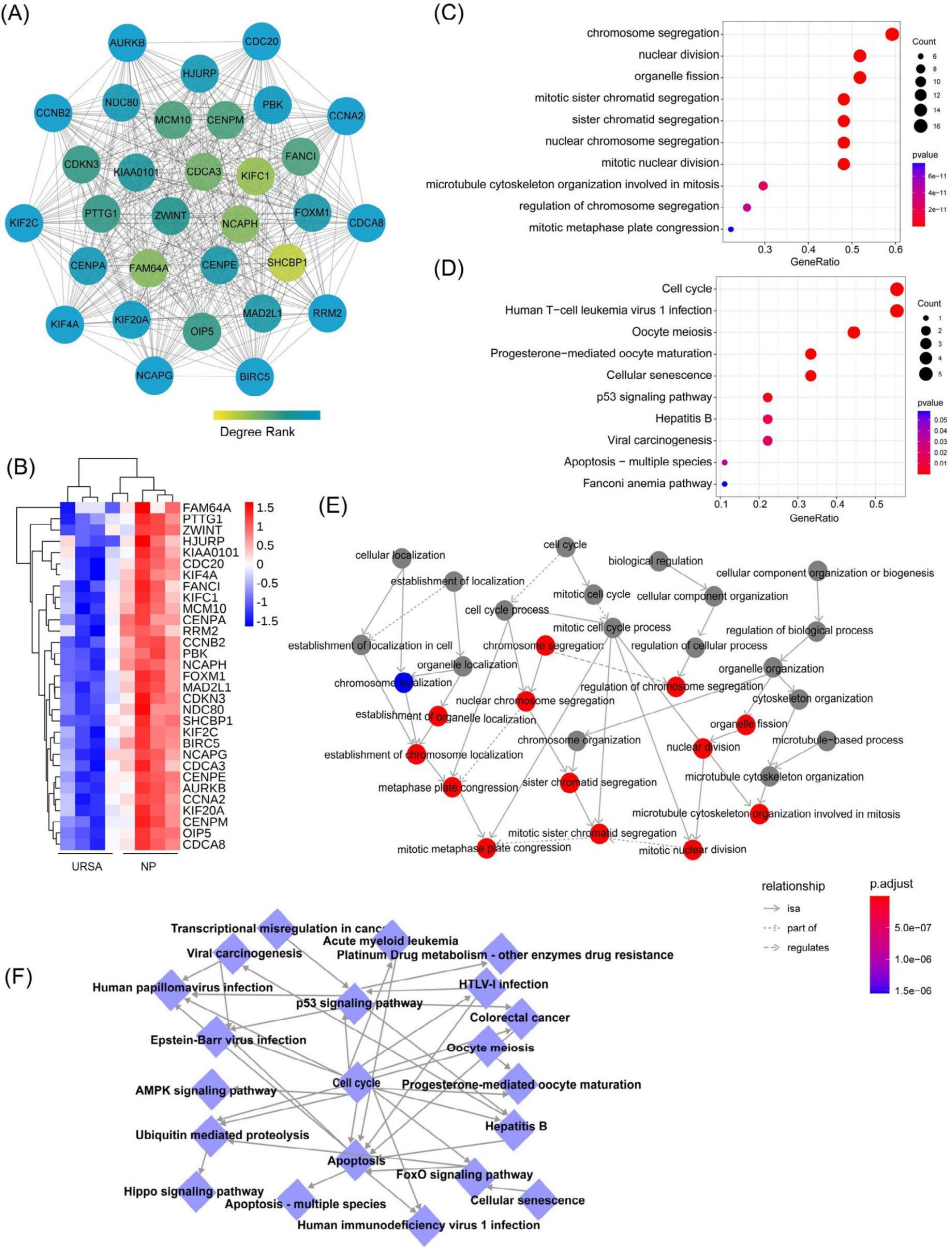
Functional enrichment analysis of hub mRNAs. **(A)** The PPI interaction network of hub dif-mRNAs. The color depth of each node correlates positively with its degree of connectivity. **(B)** The heatmaps of hub dif-mRNAs. **(C)** The top 10 significantly enriched GO terms in biological process of hub dif-mRNAs. **(D)** The top 10 enriched KEGG pathway of hub dif-mRNAs. **(E)** GO-Tree network analysis based on the interaction relationship of enriched BP terms. **(F)** Pathway-act network analysis illustrated mutual interactions between pathway terms

Considering the importance of hub dif-mRNAs in the network, we disclose hub dif-mRNAs by cytoHubba app. The nodes with high degree score can be regarded as a key node of the network. With a degree greater than 20, a total of 31 hub dif-mRNAs were identified and picked out from the PPI network (Fig. 5A). The hierarchical clustering heat map of the hub genes was constructed (Fig. 5B). All these hub dif-mRNAs were downregulated in URSA (Fig. 5B). GO analysis showed that changes in BPs of hub dif-mRNAs were mainly enriched in mitotic process, such as chromosome segregation, nuclear division, while changes in BPs CC and MF were mainly enriched in chromose, centromeric region and microtuble (Fig. 5C & Figure S4A). The chord diagram showed the hub dif-mRNAs involved in the top 10 enriched GO terms in BP, such as BIRC5, KIFC1, NCAPH, CCNB2, CDC20 (Figure S4B). KEGG analysis revealed that hub mRNAs were enriched in cell cycle, apoptosis, cellular senescence (Fig. 5D).

To further understand the core BP associated with URSA, GO-Tree analysis of BP GO terms was performed, based on their subordinate and interaction relationship (Fig. 5E). The network indicated the BPs, including regulation of chromosome segregation, nuclear division, may play a key role in URSA. To further investigate the hub pathways that may play a vital role in URSA, a pathway-act network was performed (Fig. 5F). The top pathways that showed most interactions with other surrounding pathways were cell cycle and apoptosis.

### Construction of co-dysregulated lncRNA–miRNA–mRNA networks

To explore the transcriptional regulation involved in URSA, we establish a lncRNA targeted ceRNA network with several methods to narrow down the scope of the target mRNAs that are mostly regulated by dif-lncRNAs.

**Fig. 6:**
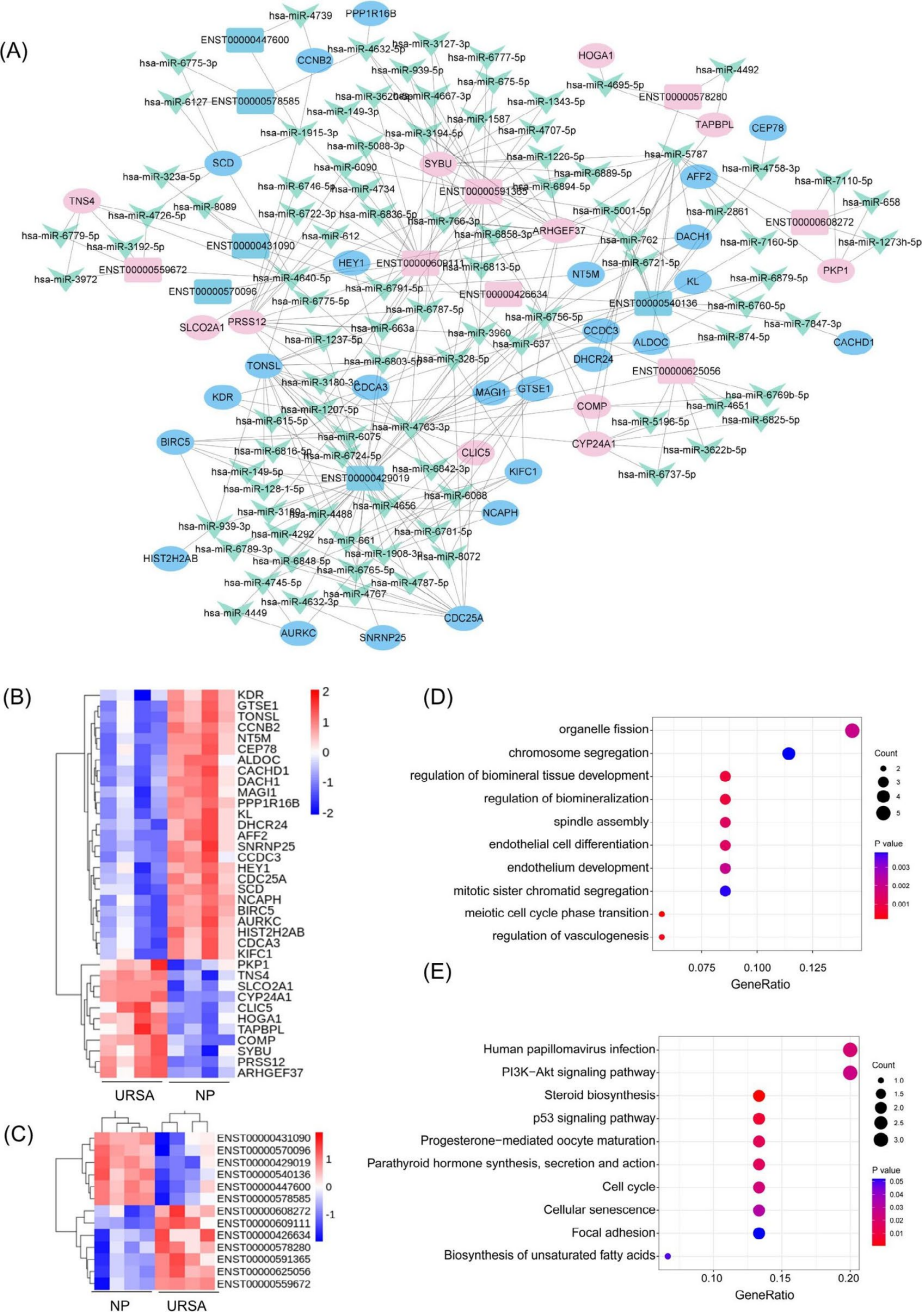
Construction of co-expression lncRNA–miRNA–mRNA networks in URSA and the functional enrichment analysis of mRNAs in ceRNA network. **(A)** Co-expression lncRNA–miRNA–mRNA networks in URSA. In this figure, lncRNA, miRNA, and mRNA were indicated by rectangle, triangle and octagon, respectively. **(B)** The hierarchical clustering heatmap of the 36 dif-mRNAs in ceRNA network. **(C)** The hierarchical clustering heat map of the 13 dif-lncRNAs in ceRNA network. **(D)** The top 10 significantly enriched GO terms in biological process of hub dif-mRNAs. **(E)** The top 10 enriched KEGG pathway of dif-mRNAs.

First, to identify co-expressed genes, we performed the Pearson correlation analysis between the dif-lncRNAs and dif-mRNAs to selected the co-expressed lncRNA-mRNA pairs. Then, the pairs with Pearson’s correlation coefficient greater than 0.97 were integrated to construct ceRNA regulatory networks to determine whether lncRNAs regulate mRNAs through miRNAs. The target mRNAs of these miRNAs were obtained using the highly reliable miRNA target prediction databases of miRanda and TargetScan. Finally, it resulted in a potential co-dysregulated lncRNA-miRNA-mRNA networks composed of 7 upregulated and 6 downregulated lncRNAs, 11 upregulated and 25 downregulated mRNAs, 96 miRNAs, and 190 edges (Fig. 6A). The heatmap of dif-mRNAs and dif-lncRNAs were shown in Fig. 6B-C.

To further clarify the biological functions of these dif-mRNAs involved in the co-dysregulated lncRNA-miRNA-mRNA networks in URSA, we performed GO functional enrichment analysis and KEGG pathway analysis for the identified dysregulated lncRNA-targeted mRNAs (fold change ≥ 2, *P* < 0.05). The top 10 enriched BP GO terms for lncRNA-regulated mRNAs in patients with URSA included cell proliferation related process, such chromosome segregation and organelle fission, and regulation of vasculogenesis (Fig. 6D). The top 10 enriched KEGG pathways of lncRNA-regulated mRNAs included cell cycle, PI3K-Akt signaling, and steroid biosynthesis (Fig. 6E). These enrichment results were consistent with the enrichment analysis associated with whole dysregulated mRNAs and hub mRNAs, which reflects the central role of these genes. These results highlighted the dysregulated cell proliferation and cell cycle process in URSA.

### Conduction of the sub-lncRNA network and key mRNAs in ceRNA network

**Fig. 7:**
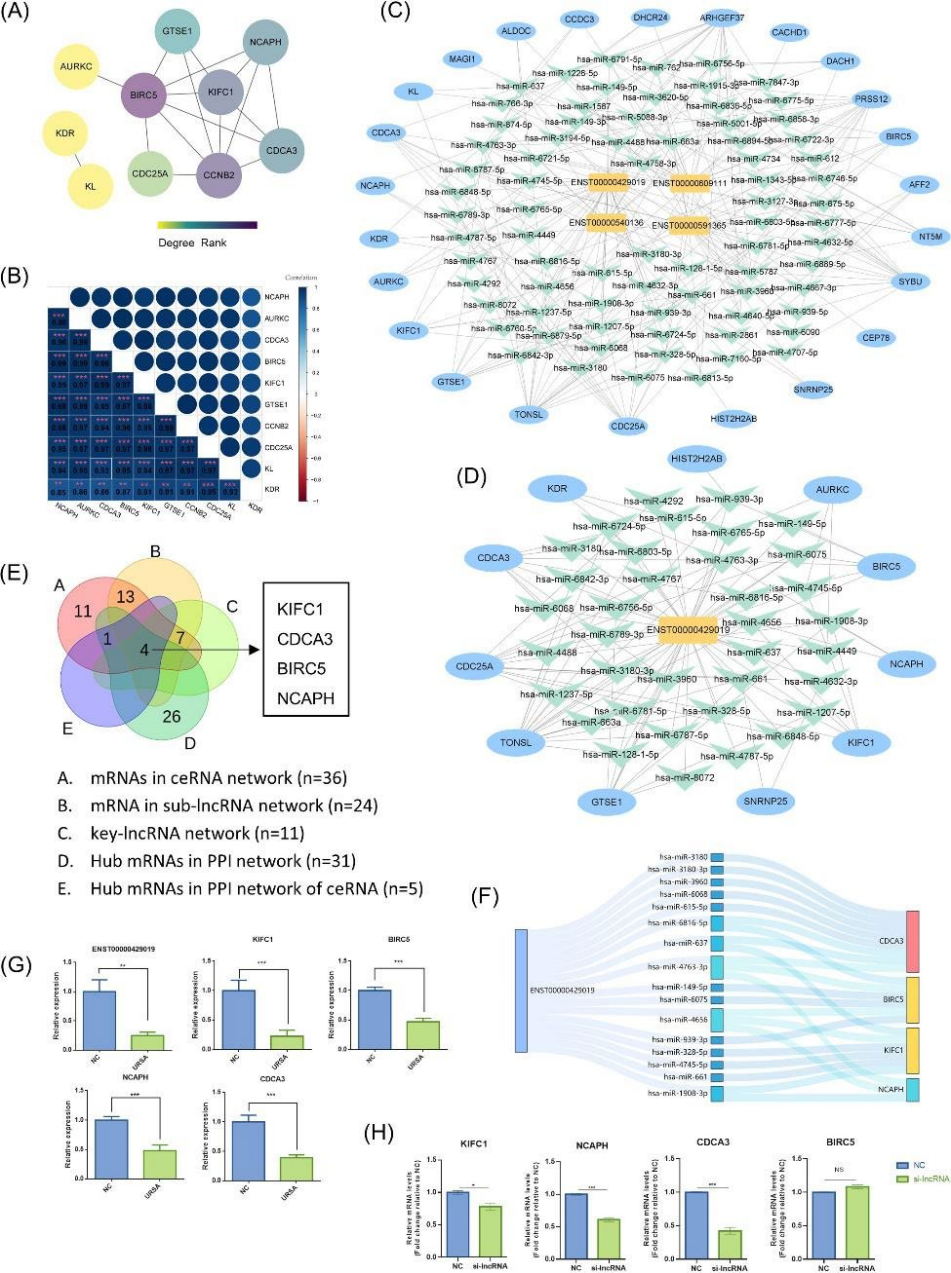
CeRNA network of key lncRNA and qRT- PCR validation. **(A)** The PPI interaction network of dif-mRNAs in ceRNA network (only significant interactions were showed). Interactions with a combine score ≥ 0.7 were considered statistically significant. Each edge links two interacting genes. The color depth of each node correlates positively with its degree of connectivity. **(B)** Pearson’s correlation analysis of 10 genes in PPI network. **(C)** CeRNA network of four hub dif-lncRNAs. **(D)** CeRNA network of key dif-lncRNA (ENST00000429019). **(E)** The key lncRNAs-mRNAs from PPI network and ceRNA network were showed in Venn diagram. **(F)** CeRNA network of key lncRNA-miRNA-mRNA in the sankey diagram. **(G)** The relative expression levels ENST00000429019 and key dif-mRNAs in key-ceRNA network were validated by real-time PCR on a sample set of 8 URSA patients and 8 controls. **(H)** The relative expression levels ENST00000429019 and key dif-mRNAs in key-ceRNA network were validated by real-time PCR when knocking down ENST00000429019 in HTR8/SVneo trophoblast cells. Statistical analysis was performed by the Mann-Whitney U test. All data are shown as the mean ± SEM. * P < 0.05

To further investigate the hub dif-mRNAs in ceRNA network at the protein level, a PPI network with 10 nodes (combine score ≥ 0.7) was established by STRING (Fig. 7A). The hub dif-mRNAs (degree ≥ 4) were BIRC5, CCNB2, KIFC1, CDCA3, NCAPH. The correlation of dif-mRNAs in ceRNA network was given in Fig. 7B.

In order to identify the hub lncRNAs in the lncRNA-miRNA-mRNA network that are related to URSA, we computed the node degrees. Thus, we found that the four lncRNAs with a high node degree (> 20) were considered to be the hub lncRNAs in the regulation network. These four lncRNAs (ENST00000429019, ENST00000540136, ENST00000591365, ENST00000609111), targeted to 24 mRNA and consisted of 150 edges (Fig. 7C). Among the four hub lncRNAs, the highest degree lncRNA is ENST00000429019, which was targeted to 11 mRNAs through 36 miRNAs (Fig. 7D).

To further narrow down the hub dif-mRNAs to identify the core dif-mRNAs, we merged the hub dif-mRNAs from PPI network, dif-mRNAs in ceRNA network. As the Venn diagram showed, four key mRNAs were found (Fig. 7E). These key mRNAs were all down-regulated in URSA and targeted by ENST0000429019, including the cell cycle gene CDCA3, apoptosis associated gene BIRC5, kinesin family member KIFC1, and mitosis associated gene NCAPH. The ENST00000429019 and core dif-mRNAs were used to construct key lncRNA-miRNA-mRNA networks in the sankey diagram (Fig. 7F). In order to validate the bioinformatics analysis results, 8 paired URSA and NP placental villus samples were collected. The real-time PCR analysis results of CDCA3, BIRC5, KIFC1, NCAPH and lncRNA were consistent with the microarray data (Fig. 7G). To determine the regulation function of ENST00000429019 in trophoblast cells, siRNAs were applied to silence ENST00000429019, and the efficiency was confirmed by RT-PCR (Figure S5). After silencing ENST00000429019 in HTR8/SVneo trophoblast cells, the mRNA levels of CDCA3, KIFC1, NCAPH were significantly decreased, while the mRNA levels of BIRC5 showed no significant difference (Fig. 7H).

## Discussion

In this study, we revealed that placental villus expression patterns of mRNAs and lncRNAs are significantly altered in patients with URSA compared with the healthy controls. We construct a lncRNA-associated network of URSA based on the co-expression of dif-mRNA and dif-lncRNA, and ceRNA theory, intending to seek remarkable aspects for the hub lncRNA-associated ceRNA network.

Normal placental development is regulated by a series of complex factors, and is essential for implantation and maintaining of pregnancy. Dysregulated trophoblast invasion, proliferation and apoptosis are known to increase the risk of RSA [17]. LncRNAs were found to participate in various biological processes such as cell differentiation, proliferation, cell cycle regulation in various diseases [18, 19]. Moreover, given that lncRNAs participate in the process of early pregnancy loss and preeclampsia [20, 21], it is logical to identify novel lncRNAs involved in trophoblast cell function. With the development of high-throughput sequencing and bioinformatics, a large number of novel lncRNA were identified and allowed for the discovery of their functions. However, the transcriptional profiles of trophoblast cells under pathological conditions in URSA remains relatively limited. Therefore, the aim of this work is to gain a comprehensive understanding of the role of trophoblast cells in the process of pregnancy.

We identified 361 lncRNAs, and 347 mRNAs that were differentially expressed in URSA. Of these, 214 lncRNA and 106 mRNAs were significantly upregulated, while 147 lncRNAs and 241 mRNAs were significantly downregulated in placental trophoblast of URSA. The biological function and potential pathways of these dif-mRNAs were initially analyzed by the GO analysis, KEGG pathways and GSEA analysis. ncRNA processing and DNA replication were enriched in the GO-BP of down-regulated mRNAs, indicating the important role of ncRNA regulation in the process of URSA. It is well known that inappropriate invasion, proliferation or apoptosis of trophoblast cells play a crucial role in the occurrence and development of pregnancy loss [22–24]. Consistently, the data from our GO and KEGG analysis showed that dif-mRNAs are involved in BPs and pathways including cell cycle, DNA replication, mitotic process, apoptosis. Evidence also shows that the degradation of the ECM is essential for the trophoblast migration and invasion. Consistently, we found the KEGG pathways of dif-mRNAs enriched in ECM-receptor interaction, which may be closely related to trophoblast invasion. Moreover, GO and KEGG analysis showed that dif-mRNAs are involved in BPs and pathways enriched in cytokine-mediated signaling pathway, cytokine-cytokine receptor interaction, Th1 and Th2 cell differentiation, which is consistent with the complex crosstalk in maternal-fetal interface [25]. Interestingly, the GSEA results indicate that gene sets associated with ncRNA processing, DNA replication, cell cycle are prone to downregulation in URSA, and apoptosis, cytokine-cytokine receptor interaction and ECM-receptor interaction are prone to upregulation in URSA.

To identify the hub mRNAs, PPI network analysis were applied. Interestingly, the hub mRNAs were all downregulated in URSA. We annotated these genes into GO and KEGG pathways, and found they were mainly related to the cell proliferation and cell cycle process, such as chromosome segregation, nuclear division, and organelle fission. Combined with the GO-tree and KEGG pathway-act network analysis, we predict that dysfunction in proliferation or apoptosis of trophoblast cell in URSA may at least in part lead to the deficiency of placentation. These results shed some light on the role of the hub genes and their related signaling pathways in the pathological process of URSA.

Next, by using bioinformatics tools, we built a co-expression lncRNA associated ceRNA network. To date, few functional lncRNAs have been described in trophoblast cells. For example, lncRNA NEAT1 and lncRNA-HZ04 were found to participant in apoptosis of human placental trophoblasts [15]. lncRNA-HZ04 promotes BPDE-induced human trophoblast cell apoptosis and miscarriage [26]. In the present study, we discovered a series of dif-lncRNAs in trophoblast cells that are associated with URSA, which would facilitate our understanding of post-transcriptional regulatory mechanisms of URSA. Then, we predicted the potential role of dif-lncRNAs by functional annotation of targeted mRNAs based on their co-expression. In addition, in order to further elucidate the mechanism of the ceRNA network, we also constructed a PPI network and performed enrichment analysis. The results of GO and KEGG analyses reveal that dif-lncRNAs-targeted dif-mRNAs in URSA were enriched in cell proliferation related process, such chromosome segregation and organelle fission, cell cycle and regulation of vasculogenesis. The dif-mRNAs involved in these processes included cell cycle related genes, CCNB2; cell division cycle associated genes such as CDC25A and CDCA3; mitosis associated gene, KIFC1 and NCAPH; vasculogenesis regulation gene, HEY1 and KDR, and apoptosis associated gene BIRC5 and GTSE1.

Interestingly, we found that most ceRNA networks were regulated by a relatively small number of hub-lncRNAs, suggesting that these hub-lncRNA may be potential key regulators controlling the URSA related ceRNA network. The lncRNA ENST00000429019 with highest degree was singled out for further explore. Next, to reveal the hub ceRNA network and identify key mRNAs that may play important roles in URSA, we performed Venn diagrams of hub dif-mRNAs and dif-mRNAs in ceRNA network. Four genes then came out of this merge: CDCA3, BIRC5, KIFC1, NCAPH. It was worth noting that these genes were all down regulated in URSA and closely related to cell proliferation and apoptosis. Through validation in URSA placental villus and silencing ENST00000429019 in HTR8/SVneo trophoblast cells, CDCA3, KIFC1 and NCAPH were significantly decreased in URSA placental villus and HTR8/SVneo trophoblast cells when silencing ENST00000429019.

CDCA3 is a cell division cycle associated gene, and involved in cell division and protein ubiquitination. CDCA3 has been widely reported as a prognostic marker for gastric cancer [27, 28]. NCAPH (Non-SMC condensin I complex subunit H), also a regulator of cell cycle, encodes a member of the barr gene family and a regulatory subunit of the condensin complex, which is associated with mitotic chromosome and cell proliferation [29, 30]. However, the role of CDCA3 and NCAPH in URSA remains unclear. However, other two key mRNAs have been reported to be associated with pregnancy in previous studies. KIFC1 belongs to the kinesin family, which is known as a motor protein essential for mitosis and serve as motors that transport cargoes such as organelles and various important functional molecules. It has been implicated in many types of cancers, such as bladder cancer [31], disorders of the nervous system [32], hepatocellular carcinoma [33]. It is well known that the placenta plays an important role in this nutrient and waste exchange in the maternal-fetal interface [34]. A previous study has investigated that KIFC1 was mainly localized in the syncytiotrophoblast both in early and term placental samples. As its increased expression pattern in preeclampsia and diabetes, it may be involved in complex trophoblast functions and placental pathologies [35]. In our study, the lower expression of KIFC1 in URSA may lead to placental dysfunction through transport deficiency, leading to URSA. Therefore, it is reasonable to assume that these screened key dif-mRNAs might play a pivotal role in the development of URSA.

In conclusion, this study explored the molecular mechanism of URSA by revealing the mRNA and lncRNA expression pattern and constructed a co-dysregulated ceRNA network. We have reason to believe that lncRNAs in the ceRNA network can affect the occurrence and development of URSA by regulating the expression of target mRNAs. However, sample size of this study was small and the interaction between miRNA-target pairs in the ceRNA network are worth to validate in vitro and in vivo experiments. We identified hub lncRNAs and mRNAs that have not been reported in URSA before. These hub mRNAs in the ceRNA network were closely related to trophoblast cell viability, apoptosis, and cell cycle, which may play fundamental roles in the pathogenesis that may occur in URSA. Therefore, this study improves our understanding of the potentially functional lncRNAs and its associated networks in trophoblast cells with URSA. These findings pave the way to the genomic complexity of URSA and may provide an initial perspective for the pathogenesis and treatment of URSA.

## Electronic supplementary material

Below is the link to the electronic supplementary material.

Supplementary Material 1

Supplementary Material 2

Supplementary Material 3

Supplementary Material 4

Supplementary Material 5

Supplementary Material 6

## Data Availability

The data that support the findings of this study are available in the methods and/or supplementary material of this article.
