# Supplementary material for: LncRNA landscape and associated ceRNA network in placental villus of unexplained recurrent spontaneous abortion
Source: Reprod Biol Endocrinol. 2023 Jun 20;21:57. doi: 10.1186/s12958-023-01107-4 (PMC10280933; doi:10.1186/s12958-023-01107-4)
Supplement: Supplementary file 1 — Supplementary Material 1 [file 12958_2023_1107_MOESM1_ESM.docx]

**Supplementary Table 1. Clinical characteristics of the women included in this study**

|  | NP  （n=15） | URSA  (n=15) | P value |
| --- | --- | --- | --- |
| Age (year) | 28.13 ± 1.28 | 29.07 ± 0.90 | 0.5166 |
| Gestational weeks (days) | 60.80 ± 2.06 | 64.27 ± 2.17 | 0.2802 |
| Smoking status | None | None | - |
| BMI | 20.91±0.41 | 21.55±0.32 | 0.2240 |
| Previous unexplained abortion | 0 | 1-2 | <0.0001 |

*NP, normal pregnancy; URSA, unexplained recurrent spontaneous abortion.

**Supplementary Table 2. Primer sequences for real-time RT-PCR**

| Gene | Primer | |
| --- | --- | --- |
| ENST00000429019 | F | AAAGGGAAACTGAGGTCCAAGG |
|  | R | GCTGAATGACTACAAGATGAACGAG |
| NCAPH | F | GACACCTCCAACTTTTGCCC |
|  | R | AGTTCCCAACAGGTCCCACA |
| CDCA3 | F | ATCTTCTGAATTGGACTTGCCTCT |
|  | R | GGGTTTCTGTGGGCTGTCTTG |
| KIFC1 | F | CCAGGCAGCCTTACTGACTGAG |
|  | R | ACCCGGCAGAATACACGGA |
| BIRC5 | F | GCAATGTCTTAGGAAAGGAGATCA |
|  | R | AGAGAAGCAGCCACTGTTACCA |

**Supplementary Table 3.** **siRNA sequences for ENST00000429019**

|  | sense（5'-3'） | antisense（5'-3'） |
| --- | --- | --- |
| siRNA-1 | AAGUCGGACGAAGCAGUCUTT | AGACUGCUUCGUCCGACUUTT |
| siRNA-2 | CACUCGUUCAUCUUGUAGUTT | ACUACAAGAUGAACGAGUGTT |

**Supplementary Figure Legends**

**Figure S1. GO-Tree network analysis of differentially expressed mRNAs.**

**Figure S2. Functional enrichment analysis of differentially expressed mRNAs.**

(A) The top 10 significantly enriched biological process (BP), molecular function (MF) terms, cellular component (CC) terms of differentially expressed mRNAs (dif-mRNAs). (B) The top 10 significantly enriched MF terms of dif-mRNAs were showed in chord diagram. (C) The top 10 significantly enriched CC terms of dif-mRNAs were showed in chord diagram.

**Figure S3. Functional enrichment analysis of mRNAs in module 1.**

1. The heatmaps of dif-mRNAs in module 1. (B) The top 10 significantly enriched GO terms in biological process of dif-mRNAs in module 1. (C) The top 10 enriched KEGG pathway of dif-mRNAs in module 1. (D) GO-Tree network analysis of dif-mRNAs in module 1 based on the interaction relationship of enriched BP terms. (E) Pathway-act network analysis of dif-mRNAs in module 1 illustrated mutual interactions between pathway terms.

**Figure S4. Functional enrichment analysis of hub mRNAs in PPI network.**

(A) The top 10 significantly enriched biological process (BP), molecular function (MF) terms, cellular component (CC) terms of hub differentially expressed mRNAs (dif-mRNAs). (B) The top 10 significantly enriched biological process terms of hub dif-mRNAs were showed in chord diagram.

**Figure S5. Functional enrichment analysis of hub mRNAs in PPI network.**

The silencing efficiency of two siRNA for ENST00000429019 was confirmed by RT-PCR. Statistical analysis was performed by the Mann-Whitney U test. All data are shown as the mean ± SEM. * P < 0.05.
