# Supplementary figures and images for: LncRNA landscape and associated ceRNA network in placental villus of unexplained recurrent spontaneous abortion
Source: Reprod Biol Endocrinol. 2023 Jun 20;21:57. doi: 10.1186/s12958-023-01107-4 (PMC10280933; doi:10.1186/s12958-023-01107-4)

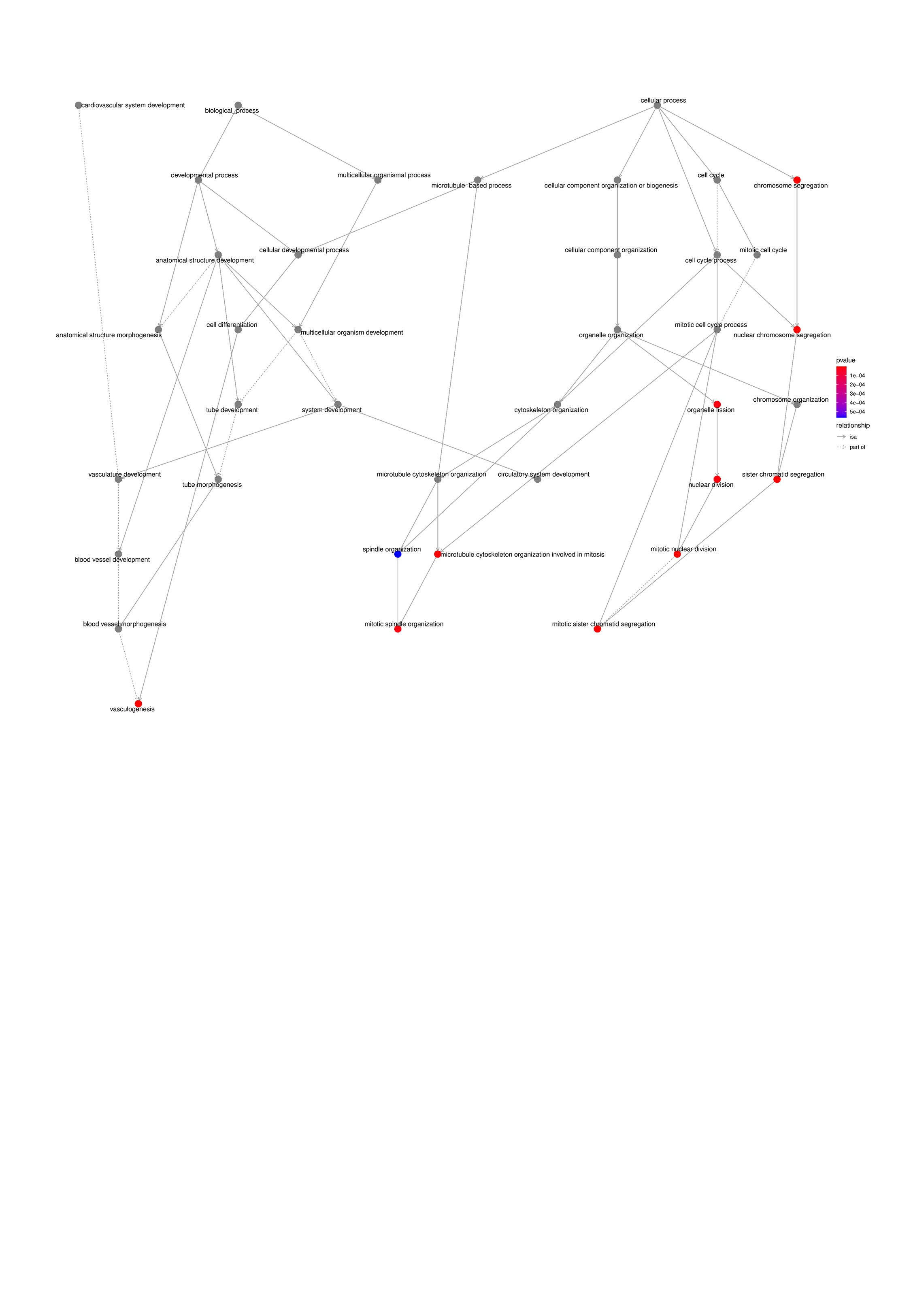

Supplement: Supplementary file 2 — Supplementary Material 2 [file 12958_2023_1107_MOESM2_ESM.jpg]

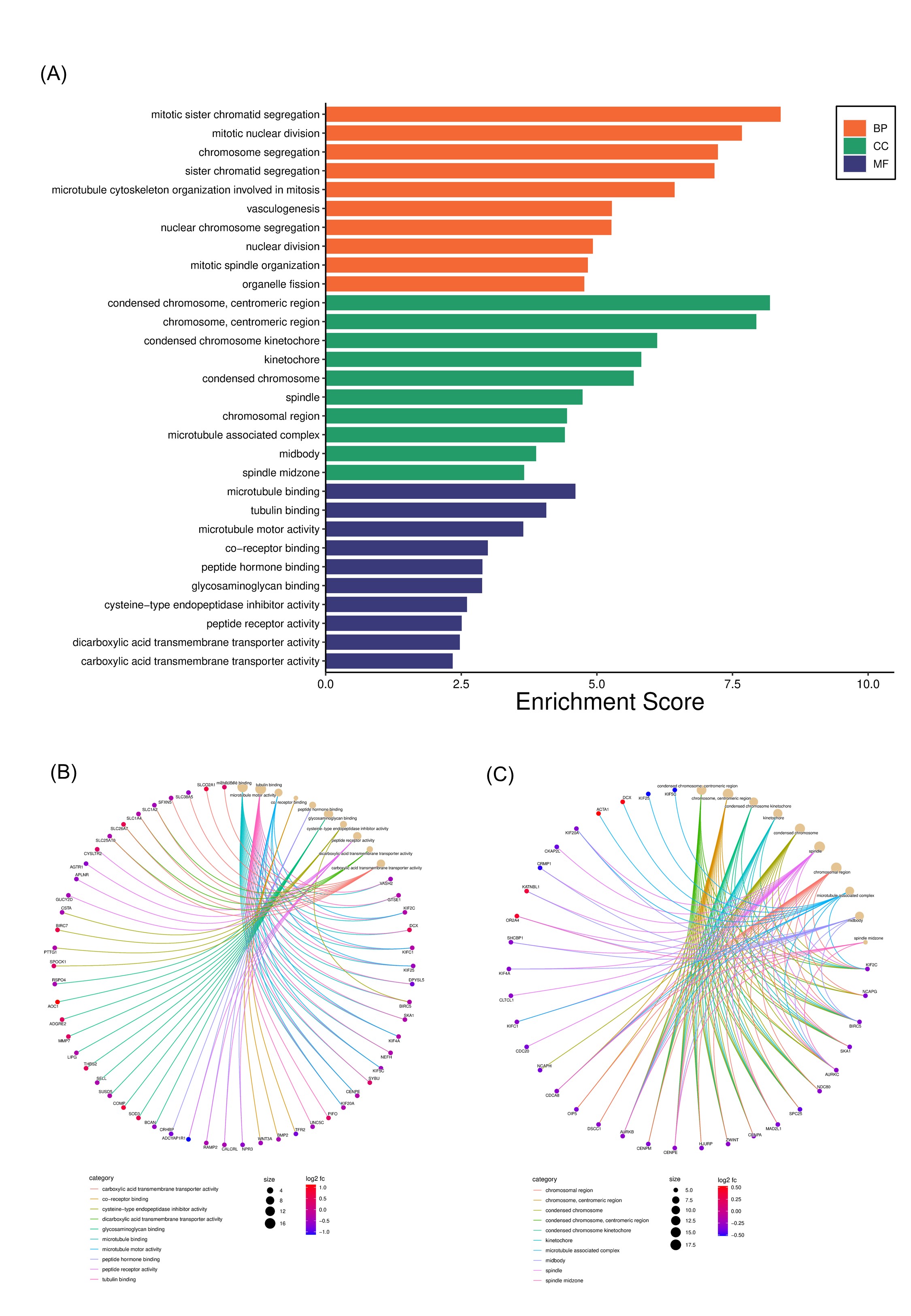

Supplement: Supplementary file 3 — Supplementary Material 3 [file 12958_2023_1107_MOESM3_ESM.jpg]

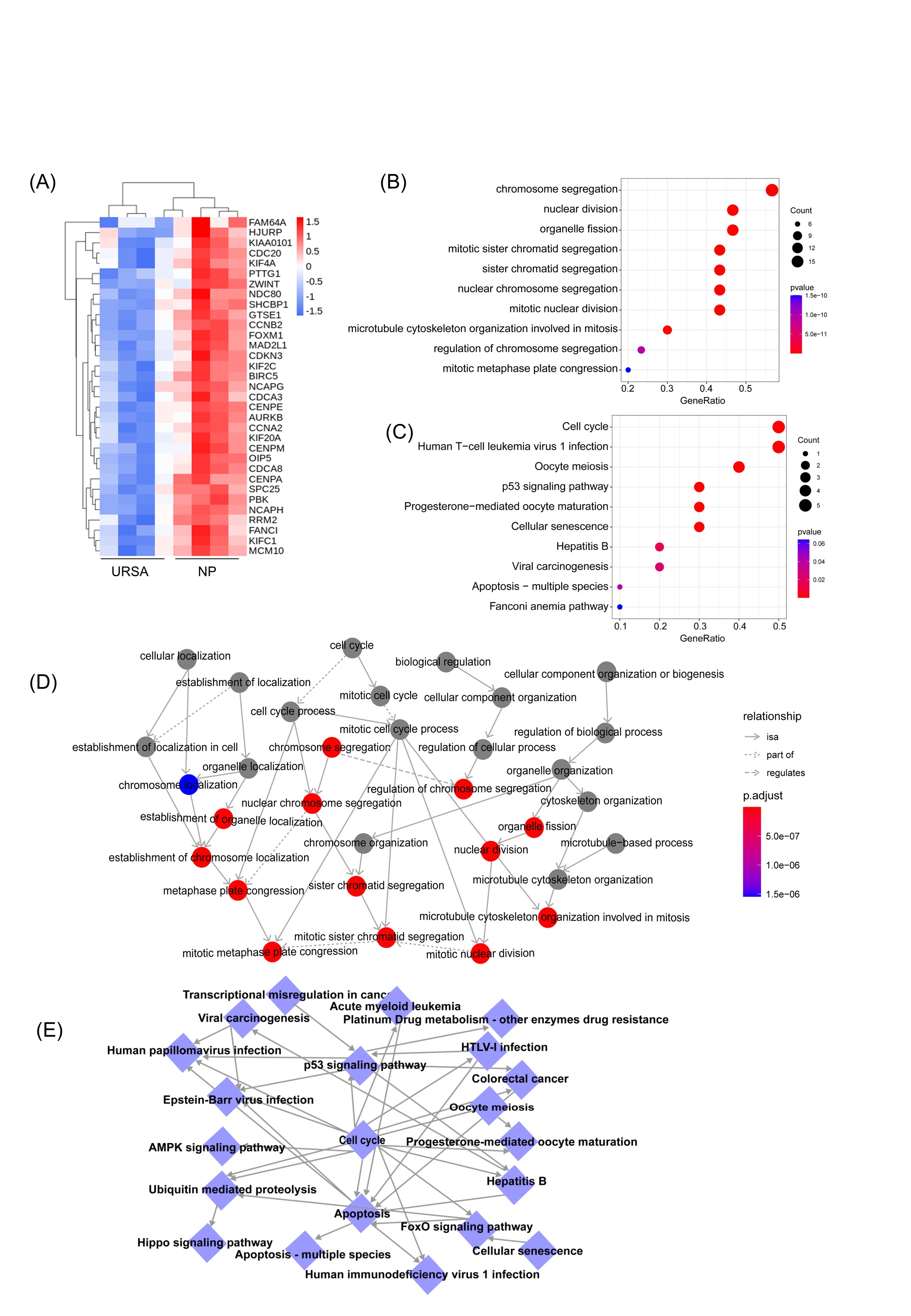

Supplement: Supplementary file 4 — Supplementary Material 4 [file 12958_2023_1107_MOESM4_ESM.jpg]

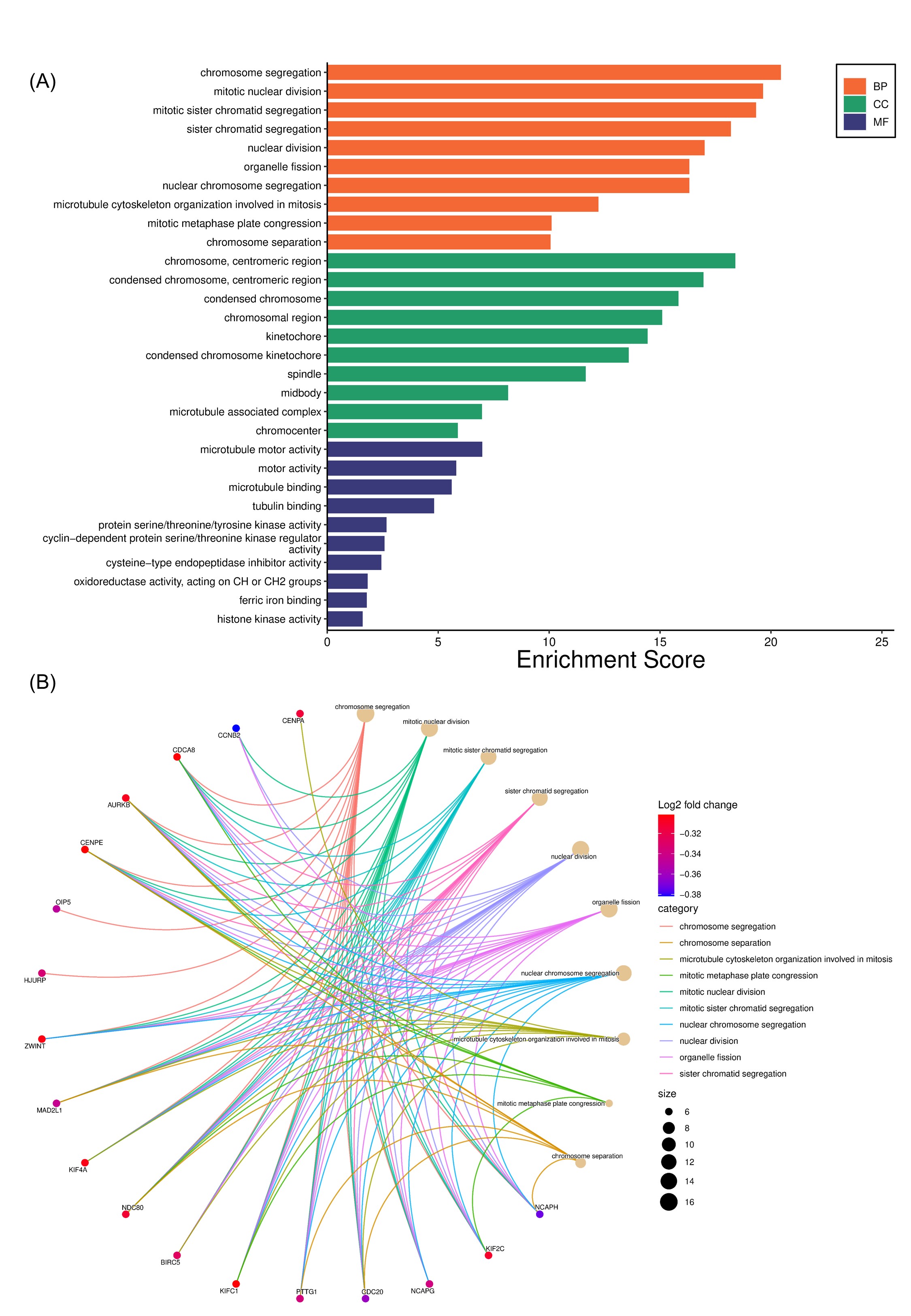

Supplement: Supplementary file 5 — Supplementary Material 5 [file 12958_2023_1107_MOESM5_ESM.jpg]

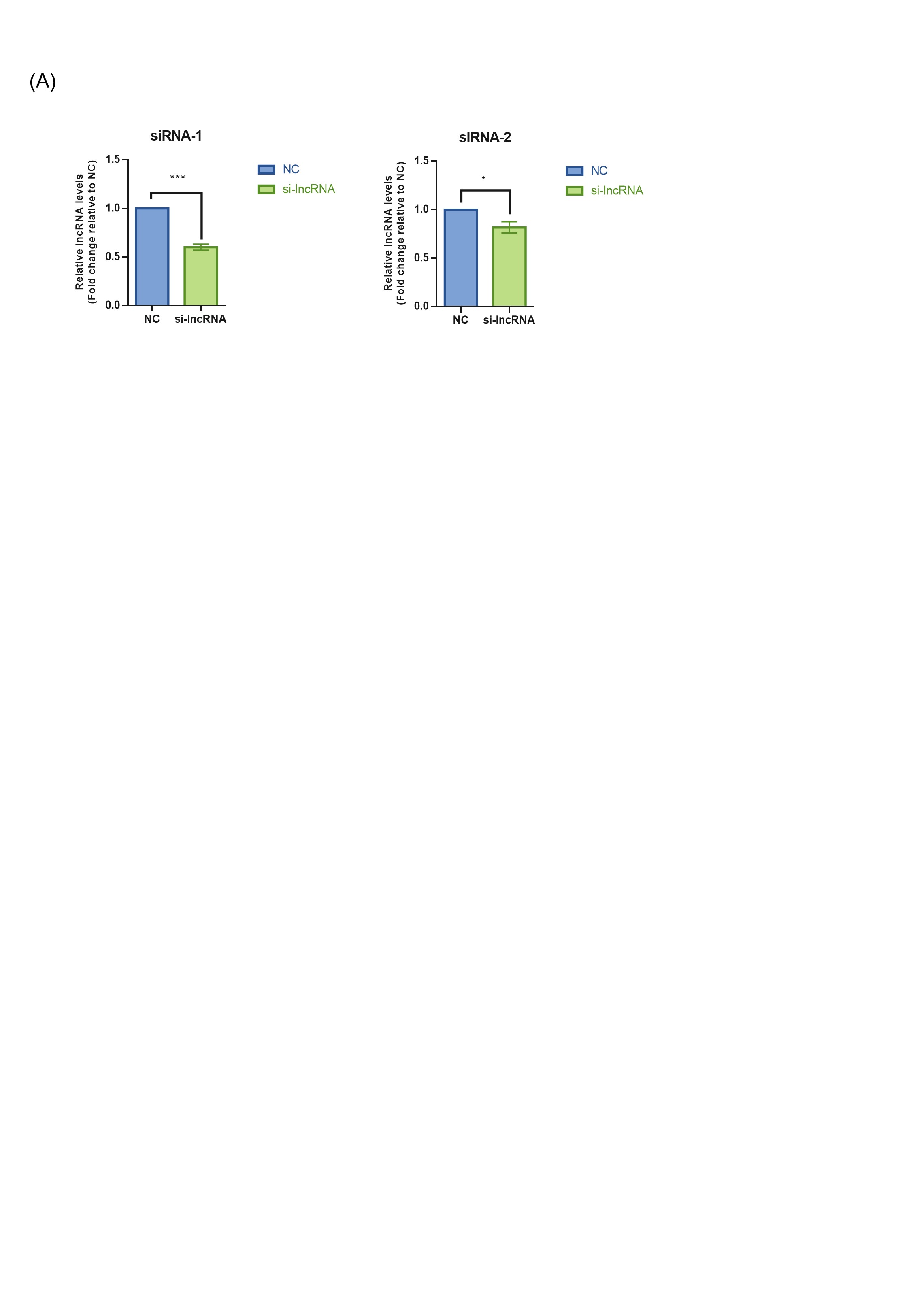

Supplement: Supplementary file 6 — Supplementary Material 6 [file 12958_2023_1107_MOESM6_ESM.jpg]
